# Telocytes in human liver fibrosis

**DOI:** 10.1111/jcmm.12542

**Published:** 2015-02-08

**Authors:** Siyi Fu, Fei Wang, Yan Cao, Qi Huang, Junjie Xiao, Changqing Yang, Laurentiu M Popescu

**Affiliations:** a Regeneration and Ageing Lab and Experimental Center of Life Sciences, School of Life Science, Shanghai UniversityShanghai, China; b Innovative Drug Research Center of Shanghai UniversityShanghai, China; c Division of Gastroenterology and Hepatology, Digestive Disease Institute, Tongji Hospital, Tongji University School of MedicineShanghai, China; d Department of Surgery, Tongji Hospital, Tongji University School of MedicineShanghai, China; e Department of Ultrastructural Pathology, Victor Babes National Institute of PathologyBucharest, Romania; f Department of Advanced Studies, “Victor Babes” National Institute of PathologyBucharest, Romania

**Keywords:** telocytes, liver, fibrosis, CD34, vimentin, PDGFR-α, β, multiple sclerosis, stellate cells

## Abstract

Liver fibrosis is a wound-healing response which engages a variety of cell types to encapsulate injury. Telocyte (TC), a novel type of interstitial cell, has been identified in a variety of tissues and organs including liver. TCs have been reported to be reduced in fibrotic areas after myocardial infarction, human interstitial wall's fibrotic remodelling caused either by ulcerative colitis or Crohn's disease, and skin of systemic sclerosis. However, the role of TCs in human liver fibrosis remains unclear. Liver samples from human liver biopsy were collected. All samples were stained with Masson's trichrome to determine fibrosis. TCs were identified by several immunofluorescence stainings including double labelling for CD34 and c-kit/CD117, or vimentin, or PDGF Receptor-α, or β. We found that hepatic TCs were significantly decreased by 27%–60% in human liver fibrosis, suggesting that loss of TCs might lead to the altered organization of extracellular matrix and loss the control of fibroblast/myofibroblast activity and favour the genesis of fibrosis. Adding TCs might help to develop effective and targeted antifibrotic therapies for human liver fibrosis.

## Introduction

Liver fibrosis, primarily as a result of chronic damages to the liver such as chronic viral hepatitis and fatty liver diseases associated with obesity, is a worldwide burden 1–3. Liver fibrosis can further progress to cirrhosis, an advanced stage of liver fibrosis, leading to a major cause of morbidity and mortality worldwide 4–6. This fibrotic response causes all the complications of end-stage liver disease, including portal hypertension, impaired metabolic capacity, synthetic dysfunction and ascites 1. Unfortunately, currently no standard treatment for liver fibrosis is available and no appropriate antifibrotic drugs has emerged to be effective in human 6–8.

Telocytes (TCs) are a novel type of interstitial cell identified in a variety of tissues and organs including liver 9–19. Telocytes are completely different from fibroblasts by their distinctive immunophenotypes 9,10,20, microRNA profiles 21, gene features 22,23 and proteome signatures 24. The striking ultrastructural hallmark of TCs is their very long and thin prolongations called telopodes (Tps) with dilatations (podoms) and thin segments (podomers) 9,10,25. The presence and the conformation of TCs was confirmed by the most advanced microscopic technology available (FIB-SEM Tomography) 26. By heter- and homocellular junctions or extracellular vesicles secretion, TCs participate in intercellular signalling, tissue remodelling, renewal and regeneration 9,10.

Telocytes have been reported to be reduced in myocardial infarction, especially in fibrotic areas 27. In addition, TCs were progressively lost from the skin, gastric wall, the myocardium and the lung of systemic sclerosis patients 28,29. Moreover, the number of TCs is decreased resulting in bowel architectural dysmotility and derangement during human intestinal wall's fibrotic remodelling affected either by ulcerative colitis or Crohn's disease 30,31. We recently reported the existence of hepatic TCs in the Disse space with a similar density in the four hepatic lobes and their potential role in liver regeneration was emphasized in a mouse model of partial hepatectomy 11,32. However, the role of TCs in liver fibrosis remains unclear.

In this study, based on immunofluorescence methods including double labelling for CD34 and c-kit/CD117, or vimentin, or PDGF Receptor-α, or β, we showed that in human liver fibrosis, hepatic TCs were significantly decreased, suggesting that loss of TCs might lead to the altered organization of extracellular matrix and loss the control of fibroblast/myofibroblast activity and favour the genesis of fibrosis.

## Materials and methods

### Samples

Human liver fibrosis samples were obtained from liver biopsy examination in Division of Gastroenterology and Hepatology, Digestive Disease Institute, Tongji Hospital. As healthy control tissues, samples were from patients who underwent surgery because of neoplastic pathologies. Specimens were collected at least 10 cm from the margin of the tumour. Healthy specimens were carefully chosen to be sure no inflammatory or neoplastic infiltration occur. All samples were divided to healthy or fibrosis specimens based on histopathological examination. All the patients had signed a written informed consent form and the Institutional ethical committees approved this study. The investigation conforms to the principles that are outlined in the Declaration of Helsinki regarding the use of human tissues.

### Haematoxylin–eosin and Masson's trichrome staining

Liver samples were fixed in 4% paraformaldehyde in PBS, and were embedded in paraffin, and sectioned at 5-μm thick. Haematoxylin–eosin and Masson's trichrome staining were performed with standard procedures to investigate liver histological and fibrotic changes.

### Immunofluorescent staining

For investigate the changes in TCs in human liver fibrosis, double immunofluorescent staining for CD34/PDGFR-α or CD34/PDGFR-β or CD34/Vimentin or CD34/c-Kit was used as our previously reported 32. In brief, 6-μm-thick frozen sections were mounted on Superfrost Plus slides (Shitai, China) and were fixed in 4% paraformaldehyde containing 0.05% Triton-X-100 for 20 min. After three times wash with PBS, sections were pre-incubated for 1 hr in 5% Bovine serum albumin (BSA). After that, sections were incubated overnight at 4°C with mouse monoclonal anti-CD34 (1:100, ab54208; Abcam Cambridge, UK) and Rabbit polyclonal to PDGF Receptor-alpha (1:100, 3938-1; Epitomics Cambridge, UK) and then sections were exposed for 2 hrs to goat antimouse labelled with Rhodamine (1:200, 115-165-166; Jackson West Grove, Pennsylvania, USA) and donkey anti-rabbit labelled with FITC secondary antibodies (1:200, 711-545-152; Jackson). Finally, sections were stained with DAPI (ProLong® Gold, Life Technology Carlsbad, CA, USA). The same protocol was used in Rabbit monoclonal to PDGF Receptor-beta (1:100, 1469-1; Epitomics), Rabbit monoclonal to Vimentin (1:100, 2707-1; Epitomics) and Rabbit polyclonal anti-c-kit (1:100, ab5506; Abcam). Each sections was randomly chosen from 20 images (400×) of the central area using confocal laser scanning microscope (LSM 710; Carl Zeiss MicroImaging GmbH, Jena Germany) and double immunofluorescent staining was merged using Zen 2011 software (Carl Zeiss MicroImaging GmbH). Counting was performed by two independent observers blinded to the sample classification. The density of TCs was expressed as average TCs number in these 20 images.

### Statistical analysis

All analyses were evaluated using SPSS 19.0 International Business Machines Corporation, Armonk, NY, USA. Data are expressed as mean ± SD and were analysed by using the Student's *t*-test for independent samples. *P*-values that were less than 0.05 were considered to be statistically significant.

## Results

**Fig 1:**
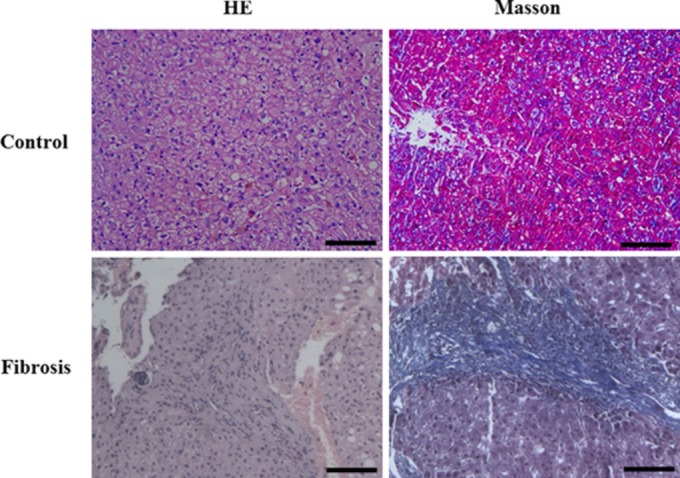
Haematoxylin–eosin and Masson's trichrome stained sections of human liver. The liver section stained with Masson's trichrome from a control, compared with a liver fibrosis patient showed significant deposition of extracellular matrix proteins. Scale bar + 100 μM.

The histopathological changes in all liver samples in this study were determined by haematoxylin–eosin and Masson's trichrome staining. In healthy liver stage group, hepatocytes had a radial array with clear central veins without occurrence of inflammation, necrosis and fibrosis (Fig.1). In fibrosis samples, obvious collagen deposition, necrosis and inflammation cells' infiltration could be identified in liver interstitial (Fig.1). The information for all participants is shown in Table1. Based on the microscopic examination results obtained from haematoxylin–eosin and Masson's trichrome staining, liver specimens were categorized in a healthy stage (*n* + 4) or a fibrosis phase (*n* + 9).

**Table 1:** Clinical characteristics of patients for liver biopsy

Case	Age	Gender	Relative liver disease	Masson*
1	50	Male	Idiopathic portal hypertension	++
2	40	Female	Medicamentous liver lesion	+
3	60	Male	Hepatic insufficiency	++
4	66	Female	Autoimmune hepatitis	+++
5	31	Male	Hepatic insufficiency	+
6	41	Female	Autoimmune hepatitis	++
7	67	Female	Hepatic lesion	++
8	55	Male	Alcoholic hepatitis	+
9	39	Male	Hepatic insufficiency	++
10	54	Male	Cholelithiasis	-
11	66	Male	Hepatic haemangioma	-
12	47	Female	Hepatic haemangioma	-
13	57	Male	Cholelithiasis	-

* Trichrome staining for fibrosis.

**Fig 2:**
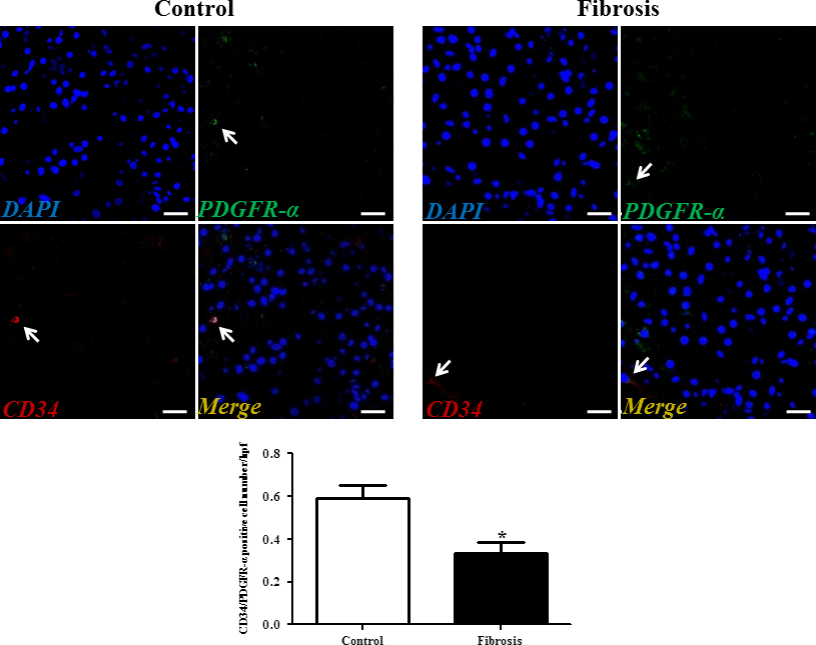
PDGFR-α/CD34 double-positive telocytes are decreased in liver fibrosis. Double immunofluorescence labelling for CD34 (red) and PDGFR-α (green) with DAPI (blue) counterstain for nuclei. Telocytes (TCs) are CD34 and PDGFR-α positive. Scale bar + 50 μm. Quantitative analysis of PDGFR-α/CD34 double-positive TCs in sections of liver from controls and liver fibrosis patients. Data are represented as mean ± SD telocyte number per high-power field (hpf). *P < 0.05, compared to controls.

**Fig 3:**
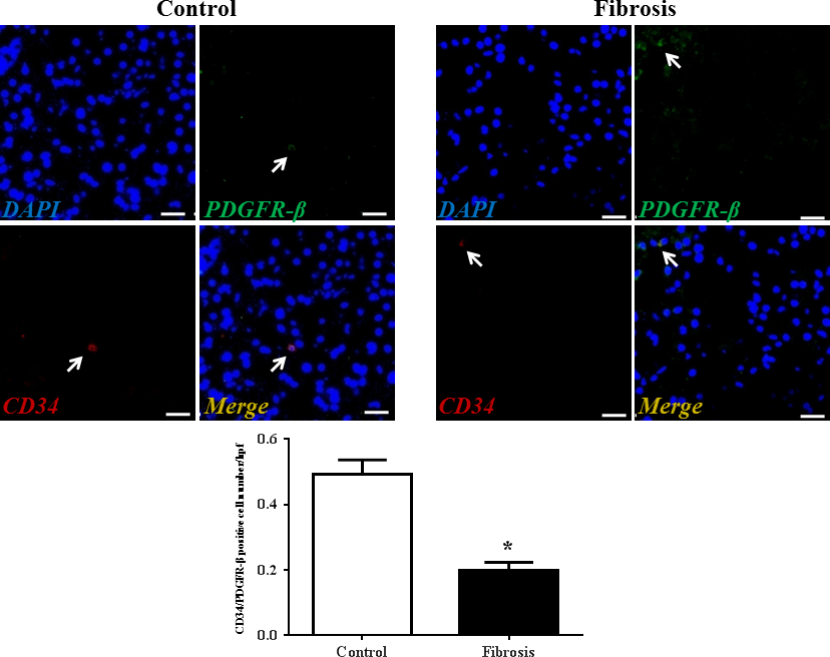
PDGFR-β/CD34 double-positive telocytes are decreased in liver fibrosis. Double immunofluorescence labelling for CD34 (red) and PDGFR-β (green) with DAPI (blue) counterstain for nuclei. Telocytes (TCs) are CD34 and PDGFR-β positive. Scale bar + 50 μm. Quantitative analysis of PDGFR-β/CD34 double-positive TCs in sections of liver from controls and liver fibrosis patients. Data are represented as mean ± SD telocyte number per high-power field (hpf). *P < 0.05, compared to controls.

**Fig 4:**
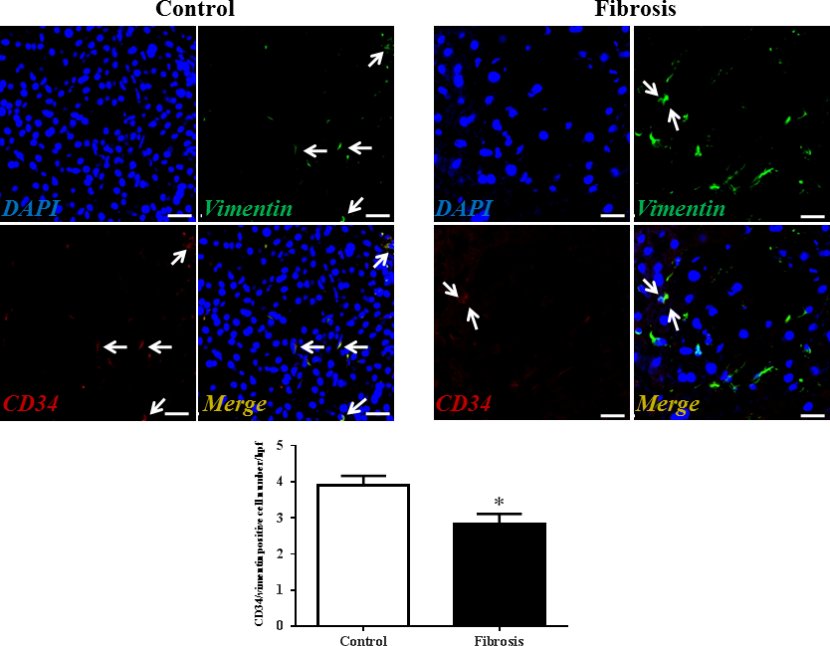
Vimentin/CD34 double-positive telocytes are decreased in liver fibrosis. Double immunofluorescence labelling for CD34 (red) and Vimentin (green) with DAPI (blue) counterstain for nuclei. Telocytes (TCs) are CD34 and Vimentin positive. Scale bar + 50 μm. Quantitative analysis of Vimentin/CD34 double-positive TCs in sections of liver from controls and liver fibrosis patients. Data are represented as mean ± SD telocyte number per high-power field (hpf). *P < 0.05, compared to controls.

**Fig 5:**
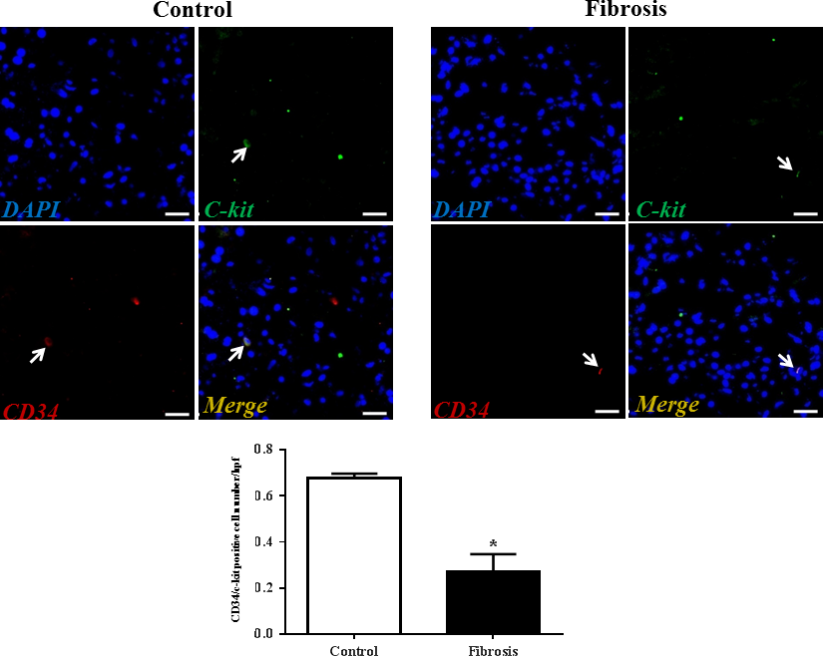
C-kit/CD34 double-positive telocytes are decreased in liver fibrosis. Double immunofluorescence labelling for CD34 (red) and c-kit (green) with DAPI (blue) counterstain for nuclei. Telocytes (TCs) are CD34 and c-kit positive. Scale bar + 50 μm. Quantitative analysis of C-kit/CD34 double-positive TCs in sections of liver from controls and liver fibrosis patients. Data are represented as mean ± SD telocyte number per high-power field (hpf). *P < 0.05, compared to controls.

Although transmission electron microscopy (TEM) examination is a golden standard for the identification of TCs, immunolabelling, especially double immunolabelling, remains a useful tool for discrimination between TCs and other cells, as well as for semi-quantitative data analysis. As we and many others have reported, here we used four different double labelling immunofluorescence methods to analyse the changes in TCs in human liver fibrosis, including double labelling for CD34 and PDGFR-α, CD34 and PDGFR-β, CD34 and vimentin, and CD34 and c-kit/CD117. Quantitative analysis of Figure2 based on CD34 and PDGFR-α double labelling showed a significant reduction in the number of TCs in the fibrotic liver. In agreement with the results based on CD34 and PDGFR-α double labelling, Figures3–5 also indicated that the number of TCs were severely reduced in liver fibrosis determined by CD34 and PDGFR-β, CD34 and vimentin, and CD34 and c-kit/CD117 double labelling.

## Discussion

This study reports a novel finding that TCs are reduced in human liver fibrosis, mirroring the recent findings as described in the colonic wall in ulcerative colitis, the terminal ileum of patients affected by small bowel Crohn's disease, and skins, gastric wall, lung and myocardium in systemic sclerosis 28–31. Our data indicate that TCs might be involved in the pathological processes of liver fibrosis.

Fibrosis is a wound-healing response which engages a variety of cell types to encapsulate injury 1,5–8. Hepatic stellate cells (HSCs) are considered as the primary source of the fibrogenic population in the liver 33. In addition, other contributions are also appreciated including portal fibroblasts, bone marrow-derived cells, circulating fibrocytes and fibroblasts from epithelial mesenchymal transition 5–8,33,34. There is now unequivocal and mounting evidence that TCs are definitely distinguished from other interstitial cells (mainly Kupffer cells and hepatic stellate cells) in liver by their location, morphology and immunophenotypes 11. The microRNA signature, differential gene expression and proteomic profiles have been established between TCs and fibroblast 21–23,35, although these signatures between TCs and HSCs still deserve explored. We here showed that TCs were decreased in liver fibrosis, further supporting that TCs are different from HSCs which is well-known to be increased in liver fibrosis. Our data based on a series of double immunoreactions clearly indicate a potential role of TCs in the genesis of human liver fibrosis. Unfortunately, because of the lack of appropriate liver samples, we could not determine the ultrastructural features of TCs by TEM and this should be acknowledged as a limitation of this study.

It remains to be determined whether the reduction in TCs precede the process of fibrosis or just a consequence of the fibrotic process. However, based on the literature, several relevant and potential roles could be proposed. First, the reduction in TCs in liver could participate in the abnormal 3D organization of the extracellular matrix as they are involved in intercellular signalling *via* either cell-to-cell contacts or shedding microvesicles or exosomes or paracrine secretion including microRNAs 36. By this, TCs might be able to control the activity of HSCs. Second, the disappearance of TCs might impair hepatocytes and stem cells mediated liver regeneration. Indeed, in recent studies, we have shown that TCs had a close spatial relationships with hepatic putative stem (progenitor) cells and TCs might influence proliferation of hepatocytes and/or the activation of hepatic stem (progenitor) cells 32. The reduction in TCs might contribute to the depletion of stem cell niches or proliferation of hepatocytes and impair liver regeneration and repair in human liver fibrosis. Further studies will be needed to reveal the functional relationship between TCs and hepatocytes/hepatic stem cells. In addition, the therapeutic utility of TCs transplantation for the treatment of liver fibrosis requires further investigation.

In conclusion, this study firstly demonstrated the decrease in TCs in human liver fibrosis based on a series of double immunoreactions. Considering the fact that intramyocardial transplantation of cardiac TCs could decreases MI and improves post-infarcted cardiac function 37, adding TCs might help develop effective and targeted antifibrotic therapies that will modify the natural history of chronic fibrosing disease.
